# Construction Notes

**Published:** 1897-05-01

**Authors:** 


### CONSTRUCTION NOTES.

### Caird Hospital for Women at Dundee Royal Infirmary.

The Caird Hospital for Women will, when completed, be one of the finest of its kind in the country, and is arranged according to the very latest ideas of hospital construction. The institution is to be built on the pavilion system, thus ensuring all the advantages of a separate hospital, yet complete separation of its departments. The hospital will be built in the grounds of the infirmary, facing the east, immediately in front of the bleaching-green park. The centre, or administrative block, will be devoted as a residence for the officials and nurses connected with the wards, and will consist of sitting-rooms and bed-rooms upstairs for nine nurses. The building has also been arranged to accommodate other six nurses if necessary. The north block, or maternity hospital, will be for the reception of women in labour, and will consist of a reception-room, where the patient will be examined before admission. On the ground floor a delivery ward, capable of holding two beds; a large ward containing four beds, together with nurses' room and kitchen. On the first floor a large ward containing four beds, similar to the one below, and three separate wards for the reception of private patients or special cases; also nurses' room and all necessary convenience. The south block will be devoted to the treatment of diseases peculiar to women, and will consist, like the maternity block, of two floors. On the ground floor are two wards containing four beds each, and between them a small special ward for a private patient, with nurse's room. Placed midway between the wards, and opening from the corridor, will be a splendid operation theatre built on the most modern plan, and embracing all the latest improvements for the safety of patients and treatment of disease. On the first floor will be a ward of four beds and three special wards, also for the reception of private or special cases. The whole hospital will contain 30 beds for patients, 17 on the south side and 13 on the north side, and will prove a most useful and important adjunct to the working of the infirmary. This splendid addition to the charities of Dundee will bring within the reach of all in need the assurance of qualified attendance at their own homes and the highest medical skill in case of need, whilst for those without homes or who otherwise desire it all the advantages of a modern hospital will be at their disposal. For the provision of such a hospital Mr. Caird is entitled to the thanks and gratitude of the community.

### New Workhouse Infirmary at Wakefield.

The foundation-stone of a new infirmary to be erected at the Wakefield Union Workhouse has been laid. The new buildings, two storeys in height, will be erected on a site of about three acres at the rear of the workhouse. The infirmary will consist of two pavilions, with an administrative block midway between them, and communicating with them by open corridors. The distance of the pavilions from one another will be 130 ft. from the administrative block. The front of the building will face due south, in which direction there is an uninterrupted view across the country, and provision is made for a future extension of the pavilions on the east and west respectively. The buildings are planned to accommodate 70 male and 78 female patients, a total of 148, and there will be large day-rooms, which could be used as sick wards when required, thus materially increasing the accommodation. The administrative block will contain rooms for the surgeon and nurses, and waiting-rooms for males and females. A mortuary and disinfecting station will be erected on the eastern portion of the site, and a new laundry near the eastern boundary. The total estimated cost of the whole scheme, including the purchase of land &c., with furnishings, is estimated to reach £36,000. Mr. William Watson, of Wakefield, prepared the plans for these handsome buildings.

